# Prehospital blood transfusion—experience from a specialized prehospital response vehicle—a retrospective cohort study

**DOI:** 10.3389/fmed.2025.1666713

**Published:** 2025-09-01

**Authors:** Frank Weilbacher, Jens Postina, Nikolai Kaltschmidt, Othmar Kofler, Maximilian Dietrich, Albrecht Leo, Markus A. Weigand, Erik Popp, Stephan Katzenschlager

**Affiliations:** ^1^Department of Anaesthesiology, Medical Faculty Heidelberg, Heidelberg University, Heidelberg, Germany; ^2^Institute for Clinical Transfusion Medicine and Cell Therapy Heidelberg, Heidelberg, Germany

**Keywords:** bleeding, trauma, transfusion, packed red blood cells, prehospital emergency medicine, hemorrhagic shock

## Abstract

**Background:**

This study evaluates the use and clinical implications of prehospital packed red blood cells administered by a specialized physician-staffed Medical Intervention Car in a German emergency medical service system capable of advanced interventions such as thoracotomy and extracorporeal cardiopulmonary resuscitation.

**Methods:**

We conducted a retrospective cohort study of all prehospital patients treated with at least one unit of packed red blood cells (pRBC) by the MIC team between August 2019 and September 2024. In the trauma cohort, we compared characteristics and interventions between those who were admitted to the hospital and those for whom resuscitation was commenced on scene. For in-hospital comparisons, patients were grouped into two categories: those who continued to receive pRBCs and those who did not. A modified blood transfusion need score (mBTNS) was retrospectively applied to assess the clinical appropriateness of transfusion.

**Results:**

A total of 57 patients received pRBCs, including 45 with traumatic and 12 with non-traumatic hemorrhage. Among trauma patients, 78% were male, 49% sustained penetrating injuries, and 56% were in traumatic cardiac arrest. There were higher rates of primary dispatch by the dispatch center in patients admitted to the hospital (43% vs. 13%; *p* = 0.048). The mean number of prehospital pRBC units transfused did not differ between those admitted and those for whom resuscitation was commenced (mean 4 [2]). Patients admitted to the hospital received fibrinogen (90% vs. 47%; *p* = 0.003), tranexamic acid (93% vs. 47%; *p* < 0.001), and Calcium (67% vs. 33%; *p* = 0.028) significantly more often compared to those who died on the scene. A lower pH and higher glucose level were significantly linked to continuous pRBC transfusion during the first 24 h after hospital admission. In the non-trauma cohort, gastrointestinal bleeding was the predominant cause (54%).

**Conclusion:**

Prehospital transfusion by a trained MIC team led to high survival rates in trauma and non-trauma patients. The frequent invasive procedures underline the need for integrated prehospital blood transfusion within advanced care. Broader adoption of structured protocols in high-acuity systems warrants prospective evaluation.

## Introduction

Despite advances in the treatment of severely injured patients, exsanguination remains one of the leading causes of death after trauma (1, 2). Campaigns, such as the Stop the Bleed campaign, promote the immediate use of tourniquets in massive external hemorrhage (3). In contrast to external bleeding, internal bleeding can only be temporarily controlled by advanced interventions such as the resuscitative endovascular balloon occlusion of the aorta (REBOA) or special surgical procedures in prehospital emergency medicine. Additionally, intravascular volume replacement is an essential pillar of therapy for hemorrhagic shock (4). Even if the cause of bleeding cannot be controlled in the field, prehospital blood transfusion (PBT) can facilitate patient stabilization for transportation, allowing for definitive hemorrhage control procedures in the hospital.

The current European Resuscitation Council (ERC) guideline on traumatic cardiac arrest (TCA) recommends activating a massive transfusion protocol (MTP) (5). However, blood products are not a standard of care in the prehospital setting, and the availability of packed red blood cells (pRBC) differs between emergency medical service (EMS) systems (6, 7). A randomized trial assessing the effect of up to two units of pRBCs and two units of lyophilized plasma compared to saline showed no difference in the primary composite endpoint (8). However, this trial has been extensively criticized concerning the selection of inclusion criteria and endpoints (8). Furthermore, only a small proportion of these patients had a TCA, limiting the applicability of these findings for patients in cardiac arrest.

This study examines the impact of PBT in an EMS setting using a specialized Medical Intervention Car (MIC). The MIC system provides advanced prehospital interventions, including thoracotomy, REBOA, and extracorporeal cardiopulmonary resuscitation (eCPR). We provide a detailed analysis of PBT cases, evaluating patient outcomes and the role of PBT in prehospital trauma care.

## Methods

### Study design and setting

This retrospective, single-centered study analyzed all MIC cases involving PBT. Ethical approval was obtained from the University of Heidelberg Ethics Committee (S 734-2024). Due to the retrospective nature of the study, informed consent was waived. All methods were performed in accordance with the relevant guidelines and regulations (Supplement Checklist STROBE) (9).

### Inclusion criteria for this study

We included all patients who received at least one unit of pRBC in the prehospital phase and were attended by the MIC. This compromises trauma and non-trauma patients. Secondary missions were excluded.

### Emergency medical service and Medical Intervention Car

The EMS system in Germany is two-tiered. To each major trauma, at least one ambulance, with one paramedic, an emergency medical technician, and one physician-staffed rapid response vehicle, is deployed. The physician’s treatment is not limited to standard operating procedures or national recommendations, allowing for individualized decisions. Furthermore, invasive procedures such as thoracostomies are included in the national training curriculum. Continuing, ‘EMS’ encompasses treatment provided by both ambulance paramedics and the emergency physician.

The MIC system, introduced in 2019, enhances prehospital trauma care by deploying two additional physicians trained in advanced interventions such as thoracotomy, REBOA, extracorporeal cardiopulmonary resuscitation, and transesophageal echocardiography. The MIC carries six units of pRBC, 4 g of fibrinogen, and 2,000 units of Prothrombin complex concentrate (PCC) (10). Since 2023, a blood warmer (°M Warmer, MEQU, Copenhagen, Denmark) has been available for prehospital transfusions. Currently, the MIC is on duty from Monday to Friday, 7 am to 5 pm, and is alerted either by the dispatch center or the EMS teams en route or on-site as a third tier.

### Prehospital transfusion algorithm

**Figure 1:**
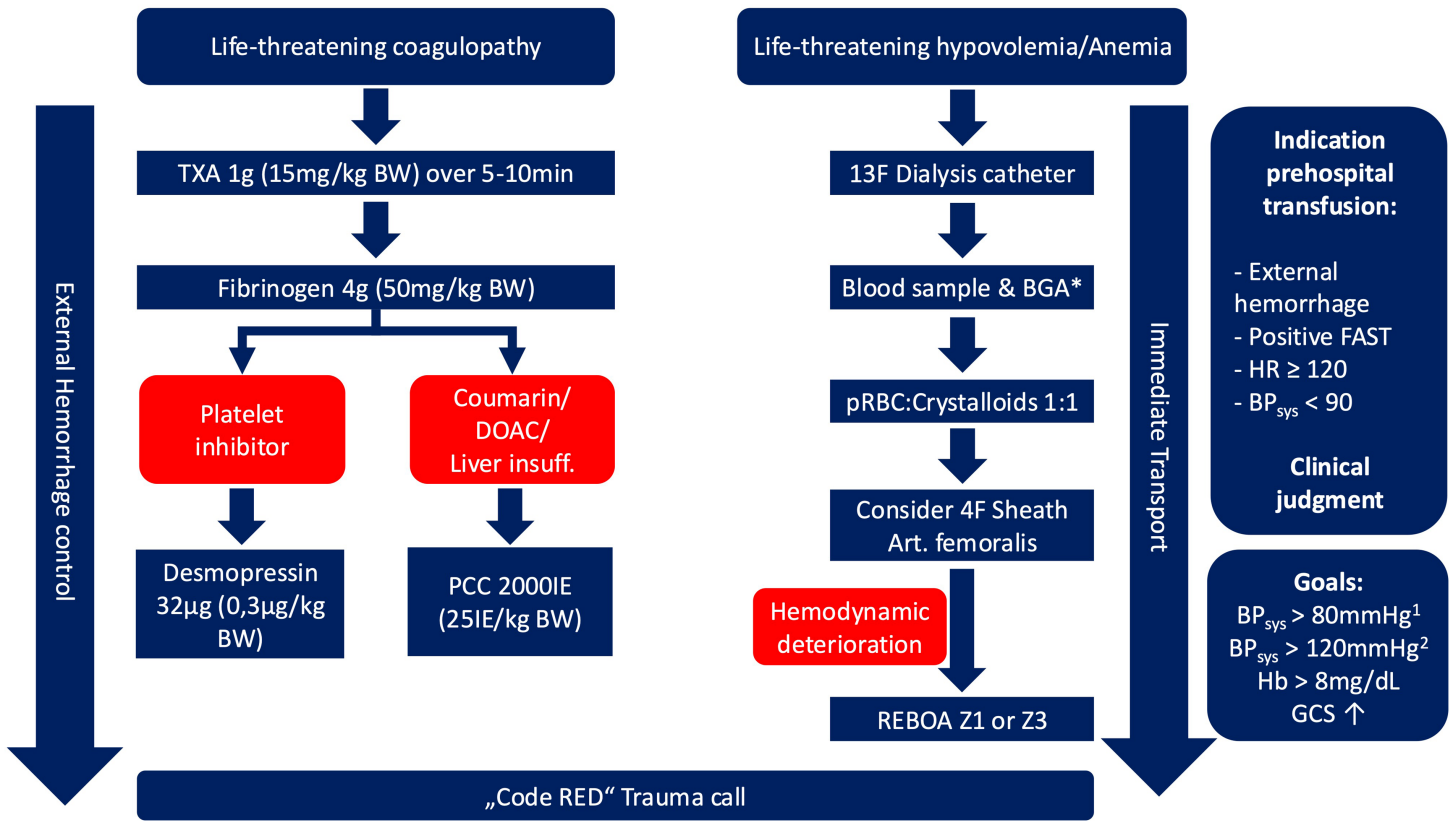
Prehospital blood transfusion algorithm. 1: in patients without presumed traumatic brain injury; 2: in patients with leading traumatic brain injury, * should not delay emergency transfusion, available since 07/2022. TXA, tranexamic acid; BW, body weight; DOAC, direct oral anticoagulants; PCC, prothrombin complex concentrate; BGA, blood gas analysis; pRBC, packed red blood cell; FAST, focused assessment with sonography in trauma; HR, Heart rate; BP, blood pressure; GCS, Glasgow coma scale.

There is no national guideline for prehospital blood transfusion in Germany. Initially based on in-hospital guidelines, the prehospital transfusion protocol has been continuously refined over the past 5 years. The protocol integrates clinical judgment, hemodynamic parameters, and validated transfusion scores (Figure 1).

### Data synthesis and statistical analysis

Electronic and paper-based documentation was used to extract data on prehospital treatment. In-hospital treatment and outcome parameters were analyzed based on the digital hospital records. We conducted a detailed descriptive analysis of all patients included in this study. Furthermore, patients were categorized into those who were admitted to the hospital, either with spontaneous circulation or under ongoing cardiopulmonary resuscitation (CPR), and those in whom resuscitation was commenced on scene. For in-hospital analysis, patients admitted to the hospital were grouped into two categories: those receiving in-hospital pRBCs and those who did not.

Prehospital time intervals were calculated from the raw data received by the dispatch center. On-scene time was defined as the time between the first EMS unit’s arrival at the roadside and the ambulance’s departure from the scene with the patient. Total prehospital time was defined as the time between the emergency call received in the dispatch center and the ambulance’s arrival at the hospital. This was only calculated for patients transported to the hospital. The functional status at hospital discharge was evaluated according to the Cerebral Performance Category (CPC), where a score of 1–2 is universally accepted as indicating good neurological function, 3–4 indicates severe neurological damage, and 5 indicates death (11).

The validated blood transfusion need score (BTNS) was assessed retrospectively as an instrument of quality assurance and adapted for traumatic cardiac arrest cases (12). A pulse of <60/min is calculated initially with a score of −4 points; however, in patients with traumatic cardiac arrest, we used the highest possible points (+3) (Supplementary Table 1). We present the analysis according to the modified BTNS (mBTNS). Blood transfusion should be considered in patients with a BTNS of >5. A BTNS of >8 indicates the need for blood transfusion.

Categorical variables were compared between the two groups using the chi-square test or Fisher’s exact test. Continuous variables were checked for heterogeneity using histograms and consequently analyzed with an independent t-test. Statistical significance is reported based on the two-sided *p*-value. The judgment of significance is reported according to the inference of the *p*-values (13). Missing data was not imputed.

## Results

**Figure 2:**
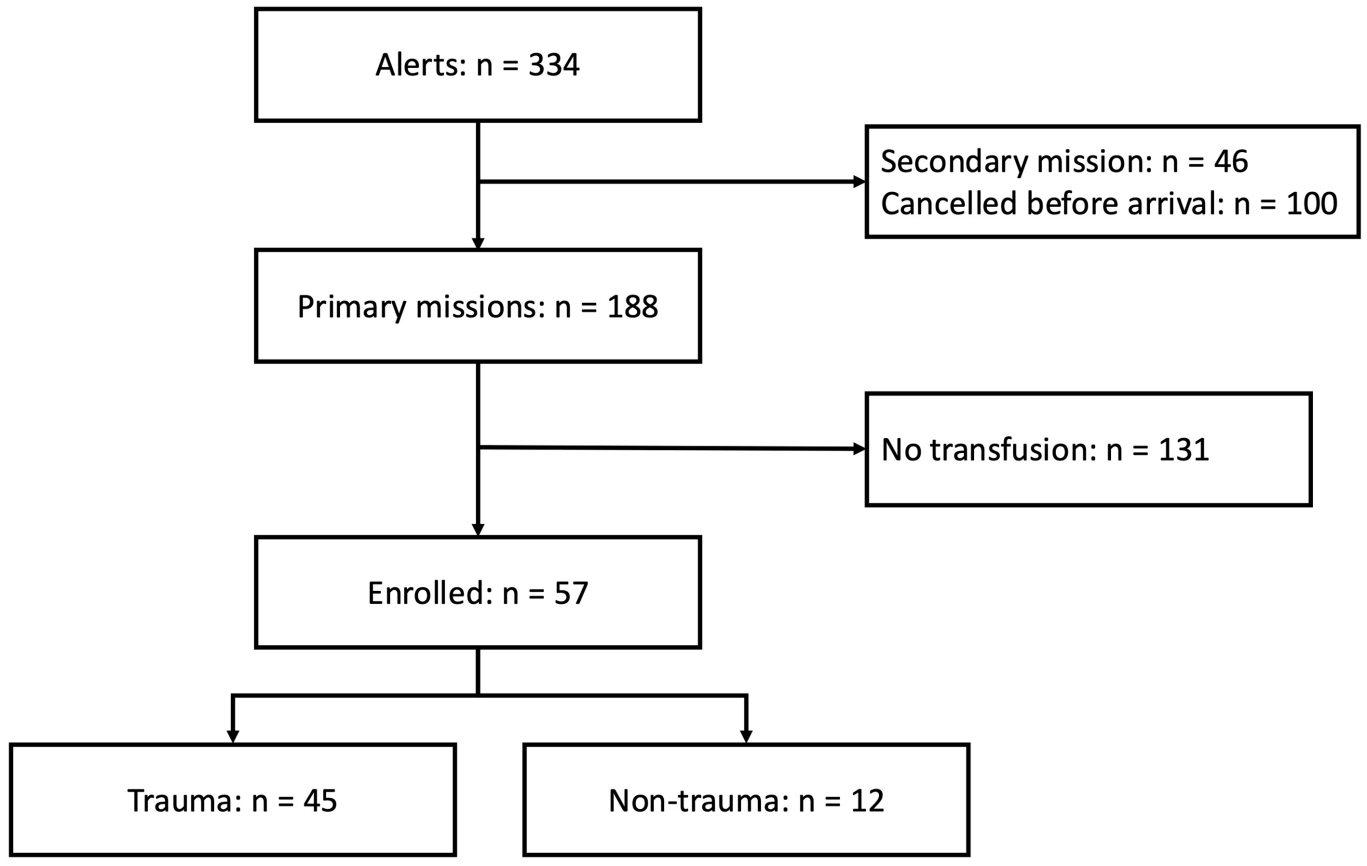
Study flowchart.

Between August 19, 2019, and September 30, 2024, a total of 57 patients were included (Figure 2).

### Trauma cohort

In the trauma cohort, the mean age was 44 years, with the majority being male (78%). Half of the patients had a penetrating trauma. Of the trauma cohort, 56% were in cardiac arrest at any point during prehospital treatment (Table 1).

**Table 1:** Demographic parameters for the trauma cohort.

Parameter	Overall (*n* = 45)	Admitted to hospital (*n* = 30)	Resuscitation commenced on scene (*n* = 15)	*p*-value
Sex, male; *n* (%)	35 (78)	22 (73)	10 (67)	1.000
Age [yrs], mean (SD)	44 (19)	40 (17)	53 (21)	0.052
Mode of dispatch, *n* (%)^*^				
Primary by dispatch center	15 (33)	13 (43)	2 (13)	0.048
Request by ambulance	29 (65)	16 (53)	13 (87)	
Location, *n* (%)				
Street	14 (31)	7 (23)	7 (47)	0.256
Home	12 (27)	9 (30)	3 (20)	
Public place	9 (20)	7 (23)	2 (13)	
Work	6 (13)	4 (13)	2 (13)	
Hospital	3 (7)	2 (7)	1 (7)	
Other	1 (2)	1 (3)	–	
Mechanism, *n* (%)				
Blunt	23 (51)	12 (40)	11 (73)	0.057
Penetrating	22 (49)	18 (60)	4 (27)	
CPR in prehospital phase, *n* (%)	25 (56)	11 (37)	14 (93)	<0.001
mBTNS, mean (SD)	10 (4)	10 (4)	12 (5)	0.242
mBTNS 0–5, *n* (%)	9 (20)	7 (23)	2 (13)	0.717
mBTNS 6–8, *n* (%)	5 (11)	3 (10)	2 (13)	
mBTNS >8, *n* (%)	31 (69)	20 (67)	11 (73)	

* 1 case missing.

yrs, years; SD, standard deviation; CPR, cardiopulmonary resuscitation; mBTNS, modified blood transfusion need score.

The mBTNS score was retrospectively assessed and not used to make the on-site decision whether to transfuse the patient. In total, 37 patients had an mBTNS score greater than 8; of these, 69% received PBT (Supplementary Table 2).

Patients receiving PBT also received invasive interventions in the prehospital phase (Table 2). All resuscitative clamshell thoracotomies were accompanied by pRBC usage. Although warmed infusion could benefit trauma patients, the blood warmer was only used in combination with pRBCs.

**Table 2:** Prehospital treatment for the trauma cohort differentiated according to total interventions performed by the MIC team and those performed by the regular emergency medical services (ambulance and regular-physician staffed response car).

Parameter	Overall (*n* = 45)	Admitted to hospital (*n* = 30)	Resuscitation commenced on scene (*n* = 15)	*p*-value
Hemostyptic gauze, *n* (%)	13 (29)	10 (30)	3 (20)	0.604
Performed by EMS, *n* (% of parent cohort)	2 (15)	1 (3)	1 (7)	
Tourniquet, *n* (%)	4 (9)	2 (7)	2 (13)	0.425
Performed by EMS, *n* (% of parent cohort)	2 (50)	2 (50)		
Endotracheal intubation, *n* (%)	34 (75)	19 (63)	15 (100)	0.008
Thoracostomy, *n* (%)	22 (49)	12 (40)	10 (67)	0.146
Performed by EMS, *n* (% of parent cohort)	9 (41)	6 (50)	3 (30)	
i.o. access, *n* (%)	18 (40)	11 (37)	7 (47)	0.717
Performed by EMS, *n* (% of parent cohort)	9 (50)	5 (45)	4 (57)	
13F Central venous Catheter, *n* (%)	30 (67)	19 (63)	11 (73)	0.738
Catecholamines, *n* (%)	40 (89)	26 (87)	14 (93)	0.068
Pelvic sling, *n* (%)	17 (38)	11 (37)	4 (27)	0.615
Performed by EMS, *n* (% of parent cohort)	2 (12)	2 (18)	–	
Blood warmer, *n* (%)	9 (20)	7 (23)	2 (13)	0.695
Fibrinogen, *n* (%)	34 (76)	27 (90)	7 (47)	0.003
PCC, *n* (%)	18 (40)	13 (43)	5 (33)	0.531
TXA, *n* (%)	35 (77)	28 (93)	7 (47)	<0.001
Performed by EMS, *n* (% of parent cohort)	7 (20)	5 (18)	2 (29)	
Calcium, *n* (%)	25 (56)	20 (67)	5 (33)	0.028
Performed by EMS, *n* (% of parent cohort)	1 (4)	1 (5)	–	
Thoracotomy, *n* (%)	13 (29)	4 (13)	9 (60)	0.005
Performed by EMS, *n* (% of parent cohort)	4 (31)	1 (25)	3 (33)	
pRBC, mean (SD)	4 (2)	4 (2)	4 (2)	0.608

PCC, prothrombin complex concentrate; EMS, emergency medical service; pRBC, packed red blood cells; EMS, emergency medical service; TXA, tranexamic acid.

Adding to the mean of 4 pRBCs in the prehospital field, 6 (SD 7) pRBCs were transfused in the first 3 h after hospital admission.

Prehospital time intervals and blood gas analyses are presented in Supplementary Tables 3, 4. Patients continuing to receive in-hospital pRBCs had a higher mBTNS (mean 13 [2] vs. 7 [4], *p* < 0.001) and received more prehospital pRBCs (mean 5 [2] vs. 3 [1], *p* < 0.001).

**Figure 3:**
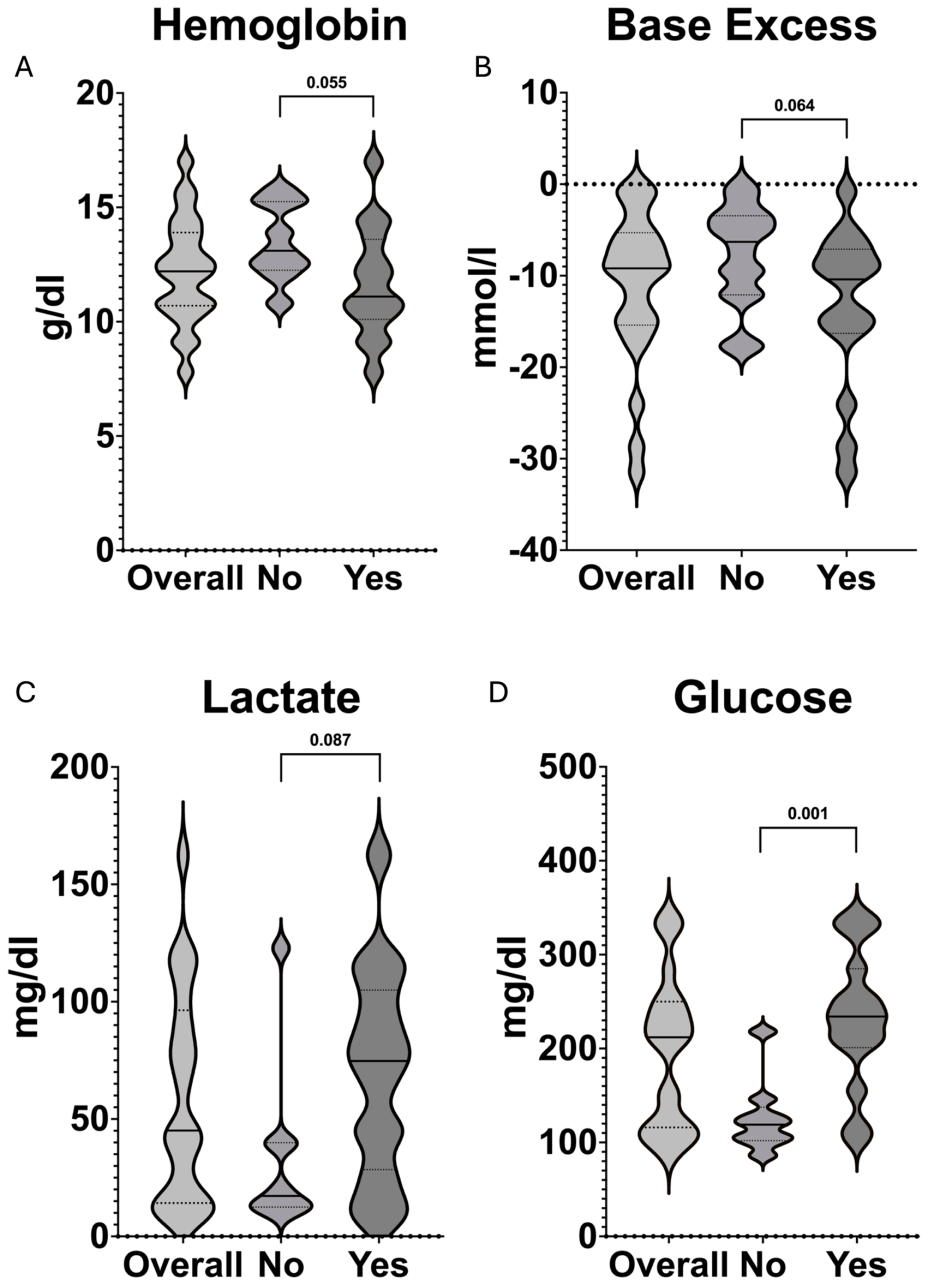
Violin plots demonstrating the distribution and comparisons between patients receiving in-hospital pRBCs (Yes) and those that did not receive further pRBCs (No) of **(A)** hemoglobin, **(B)** base excess, **(C)** lactate, and **(D)** glucose at hospital admission. The solid black line represents the median value, whereas the dashed lines indicate Q1 and Q3, respectively.

At hospital admission, there was moderate significance for pH and glucose, while base excess, hemoglobin, and lactate showed weak significance between the two groups (Table 3; Figure 3).

**Table 3:** Blood gas values at hospital arrival.

Parameter	Overall (*n* = 30)	In-hospital transfusion (*n* = 15)	No in-hospital transfusion (*n* = 15)	*p*-value
pH, median (IQR)	7.23 (7.06–7.29)	7.12 (6.99–7.26)	7.30 (7.17–7.33)	0.015
pCO_2_, median (IQR)	48 (41–59)	50 (42–63)	45 (35–50)	0.096
pO_2_, median (IQR)	68 (55–255)	77 (61–255)	63 (41–272)	0.548
Base excess, median (IQR)	−9 (−15 to −5)	−10 (−16 to −7)	−7 (−10 to −2)	0.064
Hemoglobin [g/dl], median (IQR)	12 (10–14)	11 (10–13)	13 (12–15)	0.055
Glucose [mg/dl], median (IQR)	206 (116–247)	234 (201–285)	119 (102–137)	0.001
Lactate [mg/dl], median (IQR)	44 (14–93)	74 (28–104)	17 (12–39)	0.087
HCO_3_, median (IQR)	17 (12–20)	17 (12–19)	19 (16–23)	0.108

IQR, Interquartile range.

**Figure 4:**
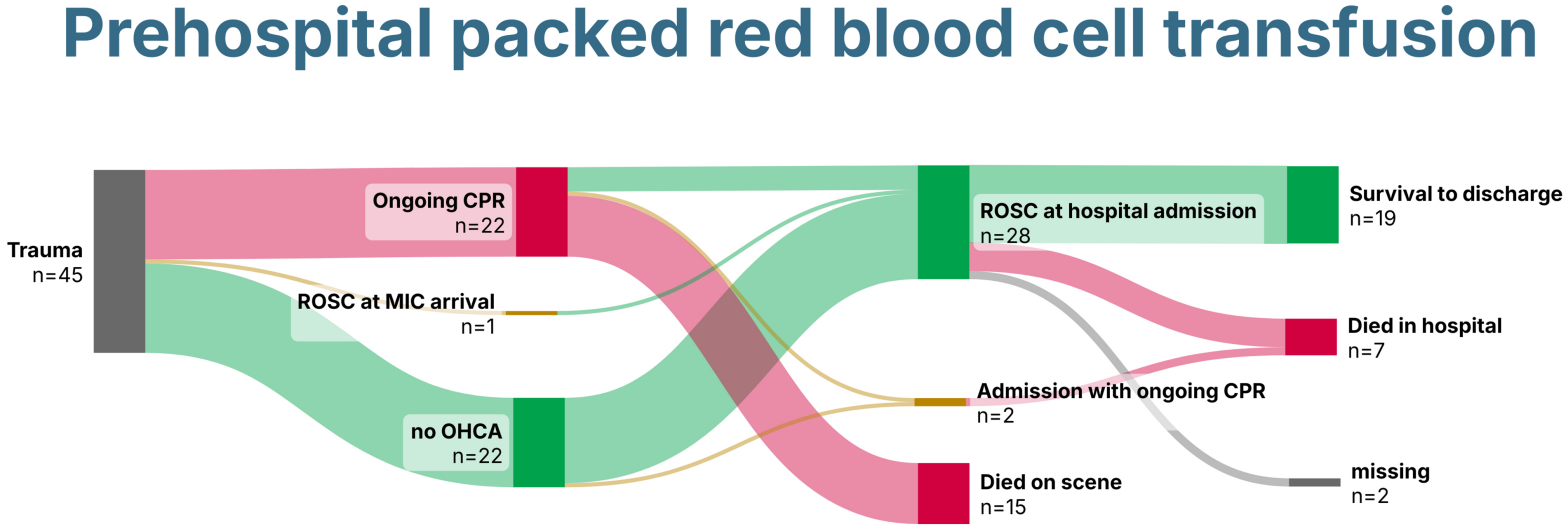
Sankey Chart demonstrating survival of patients with trauma receiving prehospital packed red blood cells. Created with SankeyArt.com. ROSC, return of spontaneous circulation; CPR, cardiopulmonary resuscitation; MIC, Medical Intervention Car.

In total, 28 patients (64%) survived until hospital admission with sustained ROSC, and two were admitted with ongoing CPR (Figure 4). Rates of survival to hospital discharge did not differ between patients who received in-hospital pRBCs and those who did not, with 8/15 (53%) compared to 11/15 (73%) (*p* = 0.420), respectively. After traumatic cardiac arrest, four patients (18%) survived to hospital discharge.

Two cases of pediatric (4 and 13 years) prehospital pRBC transfusion were included. Both experienced a TCA from a fall from height, received PBT, and underwent a clamshell thoracotomy. One had sustained ROSC upon hospital admission. However, both patients died during the in-hospital course.

### Non-trauma cohort

During the observational period, a total of 12 patients received a prehospital blood transfusion due to non-traumatic life-threatening hemorrhages. The patients were predominantly male, in their mid-fifties, and suffered mainly from gastrointestinal bleeding (Table 4).

**Table 4:** Demographic parameters for the non-trauma cohort.

Parameter	Transfusion (*n* = 12)
Sex, male; *n* (%)	11 (92)
Age [yrs], mean (SD)	56 (16)
Mode of dispatch, *n* (%)^*^	
Primary by dispatch center	2 (17)
Request by ambulance	9 (75)
Location, *n* (%)	
Street	2 (17)
Home	8 (66)
Public place	1 (8)
Other hospital	1 (8)
Medical cause, *n* (%)	
Gastrointestinal bleeding	7 (54)
OHCA with eCPR	5 (38)
CPR, *n* (%)	10 (83)
BTNS, mean (SD)	11 (4)

* 1 case missing.

yrs, years; SD, standard deviation; eCPR, extracorporeal cardiopulmonary resuscitation; CPR, cardiopulmonary resuscitation; BTNS, blood transfusion need score.

Half of the patients were also treated with fibrinogen in addition to pRBCs. Almost all patients (4/5) with i.o. access were simultaneously equipped with a 13F central venous catheter (Table 5).

**Table 5:** Prehospital treatment for the non-trauma cohort differentiated according to total interventions performed by the MIC team and those performed by the regular emergency medical services (ambulance and regular-physician staffed response car).

Parameter	Transfusion (*n* = 12)
i.o. access, *n* (%)	5 (42)
performed by EMS, *n* (% of parent cohort)	4 (80)
13F central venous catheter, *n* (%)	5 (42)
Blood Warmer, *n* (%)	3 (25)
Number pRBC, mean (SD)	4 (2)
Fibrinogen, *n* (%)	7 (58)
PCC, *n* (%)	3 (25)
Calcium, *n* (%)	4 (33)
TXA, *n* (%)	6 (50)
Performed by EMS, *n* (% of parent cohort)	3 (50)

PCC, prothrombin complex concentrate; EMS, emergency medical service; pRBC, packed red blood cells.

**Figure 5:**
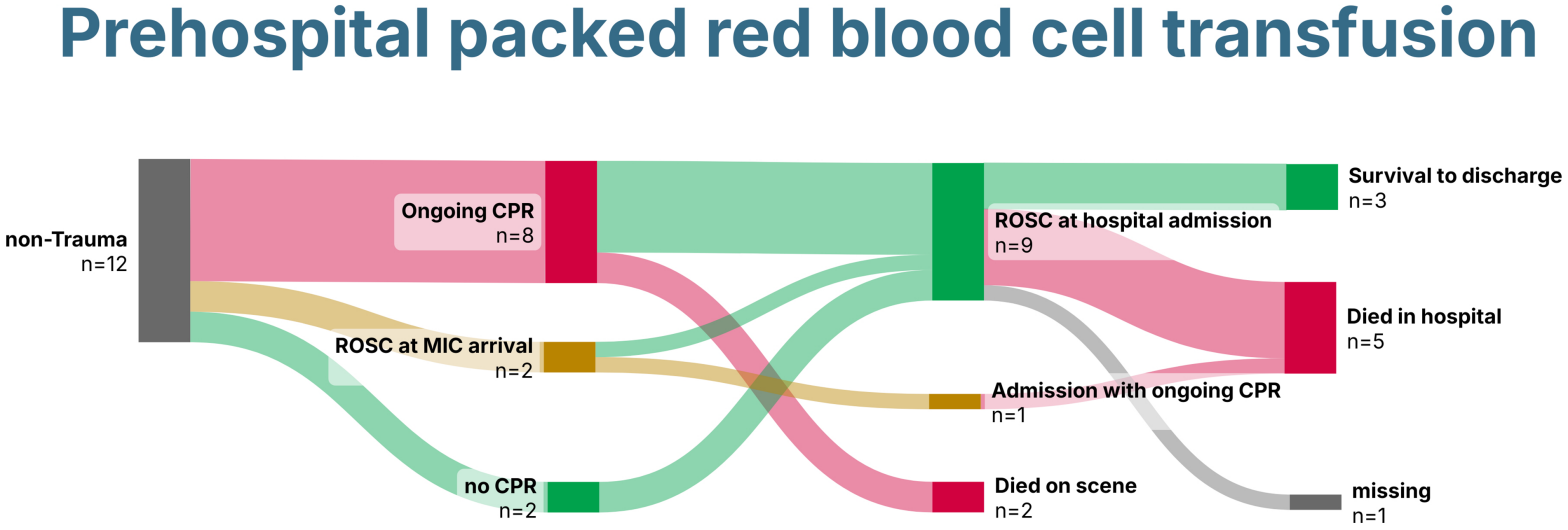
Sankey Chart demonstrating survival of patients without trauma receiving prehospital packed red blood cells. Created with SankeyArt.com. ROSC, return of spontaneous circulation; CPR, cardiopulmonary resuscitation; MIC, Medical Intervention Car.

Of the 12 patients, 10 were transported to the hospital, and four received prehospital eCPR. Three survived until hospital discharge, two with gastrointestinal bleeding, and one after prehospital eCPR, all with good neurological outcomes (Figure 5).

## Discussion

This study describes a cohort of 57 patients receiving prehospital blood transfusions for trauma and non-traumatic causes. Due to the high number of additional advanced interventions, this suggests that prehospital blood transfusion involves more than just transporting blood to the scene. There were significant differences between patients admitted to the hospital and those in whom resuscitation was commenced on scene. Those admitted to the hospital received fibrinogen, Tranexamic acid, and calcium significantly more frequently, while the mean number of pRBCs applied remained the same. This difference may be due to survivorship bias, as the dissolution of fibrinogen takes some time. A specialized prehospital team can impact short-term survival and provide an opportunity to reach definitive care (14). This requires the prompt deployment of such teams, as highlighted in the data. Among the patients admitted to the hospital, the MIC team was significantly more often dispatched primarily by the dispatch center. This has the advantage that the team arrives early on the scene, compared to a request by an ambulance.

The high number of additional interventions, such as thoracostomy or central venous access, performed by the MIC team demonstrates the complexity of these patients. The optimal number of blood products to carry in prehospital teams is still being determined; however, a mean of four pRBCs used per patient indicates that the available amount in certain EMS services might be too low to impact patient outcomes (7). Furthermore, logistics and legal requirements limit the widespread application, thereby limiting comparability between different EMS systems that carry pRBCs. Prehospital blood gas analysis revealed profound shock before blood transfusion in patients receiving PBT. Although they were not used for the decision to start prehospital transfusion, these results are in line with the current literature on the predictiveness of prehospital blood gas analysis for early in-hospital blood transfusion. Lactate and Base excess had the highest accuracy with an optimal cut-off at 4 mmoL/L and −2.5 mmoL/L, respectively (15).

Hemoglobin levels at hospital admission may appear high. Still, elevated lactate levels and a low base excess indicate ongoing hypovolemia, greatly limiting the relevance of hemoglobin as a measure for determining the appropriate transfusion volume. A multicenter study found that a point-of-care hemoglobin level of ≤12 g/dL predicted severe hemorrhage with an area under the curve of 0.77 (16). In this trauma cohort, patients who continued to receive in-hospital pRBCs had a median hemoglobin level of 11 g/dL. They received a mean of six additional pRBCs within the first 3 h after hospital admission, which corroborates this finding. Fifty per cent of trauma patients who received transfusions in the prehospital setting and were admitted to the hospital did not receive further transfusions in the first 24 h. This is consistent with evaluations from the German trauma registry of patients in whom transfusion was started in the trauma room. In these cases, non-massive transfusion (1–9 pRBCs) was also 8.5 times more common than massive transfusion (≥10 pRBCs) (17). Therefore, it could be argued that necessary non-massive transfusions were shifted from the trauma room to the prehospital setting. Future studies should further investigate this patient group to determine whether there are already signs of incipient organ damage or whether the transfusion can be omitted in individual cases. Blood glucose levels at admission were significantly higher in patients who required a further transfusion during the first 24 h of hospital stay. Previous studies showed a correlation between high blood glucose levels and mortality in trauma patients (18, 19), indicating higher injury severity in this subgroup.

Using retrospective mBTNS as an indicator of transfusion requirement, clinical judgment on the scene was accurate, with 69% of prehospital transfused patients having an mBTNS greater than 8. The mean mBTNS was 11, demonstrating the imminent need for blood transfusion. The significant difference in the mBTNS was seen in patients receiving in-hospital pRBCs. Several scores have been developed to help clinicians predict the need for blood transfusion after trauma. However, none of these scores included patients in TCA, leaving another knowledge gap for this cohort. In a European survey on the availability and indication for prehospital blood transfusion, traumatic cardiac arrest was reported to be the least indicated reason for prehospital blood transfusion. At the same time, major trauma was the main reason (6). This underlines the need for a standardized approach to treat all reversible causes of traumatic cardiac arrest. Survival was 18% in our cohort receiving prehospital blood transfusion in TCA. Despite the small number of cases, this rate appears high compared to recently published national data from the German Resuscitation Registry and the German TraumaRegister, reporting 5% survival to hospital discharge in patients resuscitated for TCA (20). Prehospital transfusion may therefore have contributed to improved survival. In our study, 22% of prehospital blood transfusions were due to non-trauma emergencies. This rate is comparable to that reported in the study by Yliharju et al., which found a rate of 31% for gastrointestinal bleeding (21). Besides gastrointestinal bleeding, this study reported the usage of pRBCs for patients requiring prehospital extracorporeal CPR. The indication for prehospital transfusion was made based on online hemoglobin values derived from the extracorporeal membrane oxygenation system, combined with blood gas analyses and clinical judgment, as well as the obvious chugging of the venous drainage cannula.

In our study, no reports of transfusion-associated adverse events were found. This low rate aligns with the current literature (22). Distinguishing between a transfusion-related adverse event and complications from the major trauma is challenging during the acute phase. This leaves a blind spot in detecting minor adverse effects and necessitates a dedicated clinical study to address this issue. Due to the exclusive use of blood group O units, adverse events related to ABO incompatibility are improbable. Retrospective compatibility testing from pre-transfusion blood samples did not reveal any relevant incompatibilities. Guidelines for prehospital transfusion should include pre-transfusion blood sampling for blood compatibility testing. This ensures rapid blood typing and ongoing use of the patient’s blood type, allowing for the detection of potential incompatibilities.

This study has several limitations: (A) Due to the retrospective nature, it can only provide associations, and prospective studies should follow to corroborate these findings. (B) A specialized team was dispatched to these cases. This may not be possible in other regions, which would limit the generalizability of our findings. (C) This study presents a real-world cohort without risk adjustment. Therefore, the survival analyses should be interpreted with caution. Future studies should focus on risk-adjusted analysis. (D) This study has a small sample size, which diminishes the significance of the analyses.

In summary, prehospital pRBC usage is associated with a high rate of invasive interventions, supporting the dispatch of an additional prehospital team for the care of bleeding patients.

## Data Availability

The raw data supporting the conclusions of this article will be made available by the authors without undue reservation.
